# Interleukin-17 as a potential therapeutic target for chronic pain

**DOI:** 10.3389/fimmu.2022.999407

**Published:** 2022-09-29

**Authors:** Xiaojuan Jiang, Ruihao Zhou, Yujun Zhang, Tao Zhu, Qian Li, Weiyi Zhang

**Affiliations:** ^1^ Department of Anesthesiology, National Clinical Research Center for Geriatrics, West China Hospital, Sichuan University, Chengdu, China; ^2^ The Research Units of West China (2018RU012)-Chinese Academy of Medical Sciences, West China Hospital, Sichuan University, Chengdu, China

**Keywords:** interleukin-17, inflammatory cytokines, neuroinflammation, neuropathic pain, inflammatory pain, cancer, chronic pain

## Abstract

Chronic pain remains to be a clinical challenge and is recognized as a major health problem with varying impacts on quality of life. Currently, the first-line therapy for chronic pain is opioids, which are often accompanied by unwanted psychoactive side effects. Thus, new and effective treatments for chronic pain are urgently needed and eagerly pursued. Inflammatory cytokines, especially interleukin-17 (IL-17), are reportedly potential therapeutic targets owing to their pivotal role in chronic pain from the neuroinflammation perspective. Recently, substantial evidence confirmed that IL-17 and IL-17 receptors (IL-17Rs) were increased in neuropathic, inflammatory, and cancer pain models. Notably, IL-17/IL-17R antibodies also reportedly relieve or cure inflammatory- and pain-related diseases. However, existing studies have reported controversial results regarding IL-17/IL-17Rs as potential therapeutic targets in diverse animal models of chronic pain. In this review, we present a summary of published studies and discuss the evidence, from basic to clinical to research, regarding the role and mechanism of action between IL-17 and diverse kinds of chronic pain in animal models and clinical patients. Furthermore, we evaluated IL-17-based therapy as a potential therapeutic strategy for inflammatory- and pain-related disease. Importantly, we also discussed clinical trials of IL-17/IL-17R targeting monoclonal antibodies. Overall, we found that IL-17 is a potential therapeutic target for chronic pain from the perspective of neuroinflammation.

## 1 Introduction

Chronic pain continues to be recognized as a major health problem with varying impacts on quality of life and increases health costs, which mainly includes neuropathic pain (NP), inflammatory pain, and cancer pain (1, 2). Chronic pain is projected to impact 37% of Americans by 2030, creating an additional economic burden of $635 billion (3, 4). The first-line therapy for chronic pain is opioids, which are often accompanied by unwanted psychoactive side effects. Thus, new and effective treatments for chronic pain are urgently needed and eagerly pursued. Recently, several research efforts have been devoted to treatment regimens for chronic pain, although there are still no effective analgesics with minimal side effects (5). Accordingly, the specific mechanisms underlying the pathogenesis of chronic pain are topics of continuous research. Neuroinflammation is an emerging target in pain relief (6). Many studies have proven that neuroinflammation is involved in multiple steps of chronic pain, such as promoting central (7) and peripheral sensitization (8).

IL-17 (also called IL-17A, CTLA8) cDNA was isolated and cloned from murine hybridomas and mainly secreted by CD4+ T cell and subtype T helper 17 cells. Th17 cells were the major source of IL-17, and other innate immune cells (e.g., eosinophils, monocytes, and neutrophils) could produce IL-17 in response to pathogens or tissue damage (9). Overall, IL-17 is produced from Th17 cells and other innate immune cells, inducing various products, including cytokines (IL-6, IL-1β, and tumor necrosis factor-alpha [TNF-α]), chemokines (CXCL1 and CCL20), and transforming growth factor-β (10).

The IL-17 family consists of six other family molecules (IL-17B, IL-17C, IL-17D, IL-17E (IL-25), and IL-17F) with structural identity (11). IL-17A and IL-17F are closely related linked genes that are usually coproduced by Type 17 cells. The IL-17 receptor family includes five members (IL-17RA to IL-17RE) that have such conserved structural characteristics as extracellular fibronectin III-like domains and Toll-IL-1R family (SEFIR) domains (12). Upon binding to the IL-17 receptor A/C heterodimeric complex, IL-17 can trigger multiple signal transduction pathways to stimulate gene transcription and increase messenger RNA stability (13). For example, the IL-17 signaling activates NF-κB, JNK, Erk1/2 and p38, CCAAT/enhancer-binding proteins (C/EBPs), Janus kinase (JAK) and phosphoinositol-3 kinase (PI3K) (12, 13).

Recently, numerous studies in humans and mice have shown that IL-17 plays a dominant role in many autoimmune diseases that affect nervous system structure and function, including multiple sclerosis, rheumatoid arthritis, psoriasis, asthma, and inflammatory bowel disease (14). Interestingly, at least some of these are attributed to the direct and indirect signaling effects of IL-17 on neurons and glial cells, such as microglia and astrocytes (15, 16). Besides, it was reported that IL-17 mainly expressed in DRG satellite glial cells expressing glutamine synthetase (GS). In contrast, IL-17Rs was detected in most neurons in DRG sections as well as in cultured DRG neurons. Recently, IL-17Rs were found to be widely expressed in small-sized mouse DRG IB4+ and CGRP+ neurons with a predominance in IB4+ neurons, and spinal cord neurons, including Somatostatin-positive (SOM)+ excitatory interneurons (expressed vesicular glutamate transporter VGLUT2) and PAX2+ inhibitory interneurons (17, 18). Thus, IL-17 and IL-17R in peripheral nervous systems may also mediate glial-neuron interactions.

IL-17 has been proven to play a critical role in neuroimmune interactions (19). For example, IL-17 released from activated microglia reportedly promotes oxygen-glucose deprivation reperfusion-induced neuronal apoptosis (20). Furthermore, IL-17 was found to have the ability to reduce the proliferation and differentiation of neural stem cells (NSCs) (21). NSCs reportedly have the potential for differentiation towards varying types of neural or glial cells (22). Notably, aside from producing IL-17, central nervous system astrocytes and microglia can also be affected by IL-17, which can lead to glial cell activation and damage, and promote the release of cytokines and chemokines, further damaging or promoting neuron growth and survival (23–25). These various effects of IL-17 may affect the cellular source and secondary release of other cytokines, and may be closely related to the target cell types.

Due to the effect of pro-inflammatory cytokines on communication between glia, immune cells, and neurons, pro-inflammatory cytokines have been implicated as integral to chronic pain development (26). In particular, IL-17 has been repeatedly verified to indirectly cause chronic pain by promoting immune cell infiltration to injured tissues (27) and releasing pro-nociceptive inflammatory factors (28), or by acting directly on neurons to regulate synaptic transmission (18). Moreover, many scientists have found that anti-IL-17 and anti-IL-17R antibodies can relieve pain (29). These studies all suggest that IL-17 plays a significant role in chronic pain initiation and progression. Recent studies also emphasize the tremendous potential of anti-IL-17/IL-17R antibodies as a potent drug for chronic pain. Hence, in this review we summarized the key evidence regarding the role of IL-17 in chronic pain.

## 2 Role of IL-17 in diverse types of chronic pain

### 2.1 Neuropathic pain

NP originates from somatosensory nervous system damage, either in peripheral or central lesions, caused by various diseases (30). The main contributing factors in NP include drug-induced peripheral neuropathy (31) and nerve injury (32). To explore the mechanisms of NP, various animal models have been established, including peripheral nerve injury (PNI), spinal cord injury (SCI), and chemotherapy-induced peripheral neuropathy (CIPN) (33) models. Previous studies have found that IL-17 expression levels were increased in these animal models (29, 34, 35), indicating that IL-17 may play a critical role in the initiation and development of NP. Herein, we review the role of IL-17 in NP caused by PNI, SCI, and CIPN.

#### 2.1.1 IL-17 and PNI

Many PNI animal models (including chronic constriction injury [CCI], partial sciatic nerve ligation [pSNL], complete sciatic transection [CST] and perineural inflammation [neuritis]) have been used to study the relationship between NP and IL-17 (36–38). Investigators explored IL-17 protein expression patterns by enzyme-linked immunoassay in the nerves of four PNI models (including CCI, pSNL, CST, and neuritis) (34) and found that on day three after modeling, only the pSNL model showed increased IL-17 expression in the sciatic nerve. In contrast, IL-17 levels increased in all four PNI models at day eight after modeling. Notably, IL-17 exerted a limited role in the acute nerve injury phase and associated acute pain, while mainly playing a vital role in the later stages of nerve injury, and is involved in the inflammatory response and development of NP. Future studies should explore the role of IL-17 in the maintenance and development of NP by blocking IL-17 activity or secretion in relation to pain behavior.

Mounting evidence suggests that IL-17 contributes to the dysregulation of immune cell infiltration and glial activation after PNI and the ensuing NP, especially in neuroinflammatory responses and pain hypersensitivity. IL-17 may also be involved in the cross-communication between IL-17 and other cytokine signaling systems in the pathogenesis of NP. IL-17 deficiency in a pSNL model significantly reduced T cell and macrophage infiltration in DRG neurons and spinal microglia as well as astrocyte activation (36). Notably, IL-17 knockout (KO) mice showed significantly reduced mechanical pain hypersensitivity, but not thermal pain hypersensitivity, suggesting that pSNL-induced NP is only partially regulated by IL-17 (36). This was also consistent with the finding that IL-17 mediated articular mechanical hypernociception in a model of antigen (methylated bovine serum albumin [mBSA])-induced arthritis in mice, suggesting that IL-17 exerts a key role in the development of mechanical abnormal pain, although to a lesser extent than that in thermal nociceptive sensitization (39). After long-term exposure to IL-17A, researchers observed that expression of transient receptor potential vanilloid (TRPV) 1 was not upregulated in DRG neurons, although TRPV4 was significantly upregulated (38). In fact, TRPV4 was considered a candidate transduction molecule for mechanical nociceptive sensitization, further indicating that the specific role of IL-17 in mechanical nociceptive sensitization is associated with TRPV4 activation and upregulation. Future studies should explore the unique role of IL-17 in the mechanical pain and thermal pain hypersensitivity with further mechanism.

Day et al. also reported similar results; IL-17 KO mice manifested obviously decreased mechanical pain hypersensitivity compared with wild-type (WT) mice, as well as decreased pro-inflammatory factors (TNF-α, IL-6, and interferon-c), anti-inflammatory factors (IL-10 and IL-13), enkephalin, β-endoglin, and porphinato (37). Moreover, the IL-17 cells related to lymphocyte activation in injured sciatic nerves and spinal cords (L4-6) were observed less in nude mice than in their heterozygous littermates, further indicating that IL-17 contributes to the nervous autoimmune reaction after nerve injury (40). Researchers have consistently proved that IL−17 promotes astrocyte and pro-inflammatory cytokine proliferation (IL−1β and IL−6) during the maintenance phase of pSNL-induced NP (41, 42). Overall, these results implicate that IL-17 can regulate the process of hypersensitivity (especially for mechanical pain hypersensitivity) by modulating inflammatory cell infiltration (T cells and macrophages), glial cell activation (microglia and astrocytes), and pro-/anti-inflammatory environments (see details above) within the injured nerve. The molecular and cellular characteristics of IL-17 in the nervous system after PNI and effects on pharmacological inhibition of IL-17 bioactivity in NP warrant further investigation.

IL-17 also reportedly responds or regulates some pain-related molecules in PNI. Furthermore, CCI-induced mechanical allodynia was reversed by inhibitory matrix metalloproteinase (MMP)-9/MMP-2, resulting in the concomitant inhibition of IL-17 and TNF-α expression in nerves (43). Moreover, acute connecting segment 1 (CS1)-containing fibronectin isoform (FN-CS1) therapy reduced mechanical allodynia and downregulated the expression of IL-17 in the injured nerve, indicating that FN-CS1 contributes to mechanical hyperalgesia *via* upregulation of Th17 cells after physical trauma (44). Interestingly, blocking IL-17 was found to ameliorate hyperalgesia 7 days after SNL and significantly reduce the expression of phosphorylated calcium/calmodulin-dependent protein kinase II (p-CaMKII) and phosphorylated cAMP-response element binding protein (p-CREB) in the spinal cord, whereas recombinant (r)IL-17A treatment resulted in opposing effects. Moreover, some experiments showed that CaMKII inhibitor KN93 not only reduced SNL- or rIL-17A-induced hyperalgesia, but also downregulated p-CREB expression levels. Results of *in vitro* data showed that KN93 can also suppress rIL-17A-induced CREB activation in primary cultured spinal neurons. IL-17A could play a major part in the maintenance of NP in the spinal cord *via* the CaMKII/CREB signaling pathway (42). Overall, IL-17 may respond to other pain-related factors such as the MMP2/MMP9 axis and FN-CS1, and may regulate the CaMKII/CREB signaling pathway to mediate NP.

#### 2.1.2 IL-17 and SCI

Among patients with SCI, 70% have chronic pain, although no effective therapy has been reported, and the pathogenic mechanisms remain unclear (45). IL-17, IL-23, IL-6, IL-21, and phosphorylated (p)-signal transducer and activator of transcription (STAT) 3 expression were markedly upregulated and peaked at 24 h after SCI, indicating that IL-17 may contribute to the promotion of spinal cord neuroinflammation after SCI *via* transcription factor STAT3 activation (46). Furthermore, CCL20 aggravated neuroinflammation caused by SCI through Th17 cell recruitment and IL-17A upregulation. Furthermore, flow cytometry results demonstrated that CCL20 antibody reduced Th17 cell recruitment 14 days after SCI (47). More importantly, IL-17 activated astrocytes and increased vascular endothelial growth factor (VEGF) expression in an SCI model *via* Janus kinase (JAK)/STAT signaling pathway activation (48). IL-17 could activate the JAK/STAT pathway by mediating a rapid tyrosine phosphorylation of the JAK family (Tyk 2, JAK 1,2, and 3) and STAT 1, 2, 3, and 4. Future studies should focus on the role of IL-17 in spinal cord neuroinflammation process *via* multiple signaling pathways, such as IL-17/STAT3, IL-17/CCL20, and IL-17/VEGF/JAK/STAT.

#### 2.1.3 IL-17 and CIPN

CIPN is a dose-limiting side effect of common chemotherapy drugs (taxanes, vinca-alkaloids, platinum compounds, bortezomib, and thalidomide) (49). As previously reported, the CIPN prevalence rate was 68.1% after chemotherapy (50). Currently, we still do not know the role of IL-17 in CIPN.

Recently, researchers demonstrated the synaptic mechanisms of IL-17 in regulating pain transmission in a CIPN model (18). IL-17 released from DRG satellite glial cells influenced synaptic transmission in the spinal cord pain circuit thereby driving CIPN. IL-17 from exogenous administration or secreted by spinal cord astrocytes may enhance excitatory postsynaptic currents (EPSCs) and reduce inhibitory postsynaptic currents (IPSCs) and gamma-aminobutyric acid (GABA)-induced currents in SOM+ neurons with IL-17R detection in superficial spinal cord horn. Specifically, IL-17R-KO in SOM+ neurons reversed paclitaxel-induced excitability of SOM+ neurons and effectively relieved CIPN. Meanwhile, overexpression of IL-17 in astrocytes directly induced mechanical hyperalgesia, similar with the previously mentioned finding that PNI induces NP. IL-17 secreted by peripheral glial cells enhanced the excitability of primary sensory neurons through IL-17R, and participated in the peripheral sensitization of CIPN by the non-lymphocyte-derived IL-17 method. The specific role of IL-17 may be the direct mechanism by which it mediates NP. IL-17 is an important signal molecule in interactions between glial cells and neurons, and may become a new target for CIPN treatment. However, reports on the molecular mechanisms underlying the interaction between IL-17R and ion channels are lacking. Further studies are needed to investigate this mechanism.

## 3 IL-17 and inflammatory pain

As an innate immune response, inflammation functions as a defense barrier in the body. The most significant symptoms are redness, swelling, heat, and pain (51). Inflammatory pain is the most common clinical symptom in inflammatory disease, and is often caused by sensitizing nociceptive neurons (52). Currently, studies have found that cytokines play essential roles in inflammatory pain, including IL-6 and IL-1β (26, 53). A growing number of studies have suggested that IL-17 has several important implications in inflammatory pain.

Researchers found that IL-17A injected into the knee joints of rats promoted long-lasting sensitization of nociceptive C fibers of the joint to mechanical stimuli and rapid phosphorylation of extracellular signal-regulated kinase (ERK). Furthermore, protein kinase B was increased in the isolated culturing of DRG neuron with long-term exposure to IL-17A, which upregulated its excitability (54). Furthermore, IL-17A-KO mice had less mechanical hyperalgesia than WT mice in an inflammatory pain model, although thermal hyperalgesia was unaffected, possibly owing to regulation of TRPV4 receptors in DRG neurons (38). These data demonstrate the special role of IL-17 in mechanical hyperalgesia, which is associated with TRPV4 activation and upregulation, as well as the phosphorylation of ERK and protein kinase B.

Many studies report that IL-17 contributes to the sensation of pain in other inflammatory-related disorders in which pain is a symptom. The incidence of abnormal pain in an experimental autoimmune prostatitis rat model was higher in rat prostate tissue with upregulated IL-17 compared with that in normal control rat tissue (55). TNF-α, IL-1β, and CXCL1 expression increased after IL-17 injection in an arthritis animal model. Notably, treatment with MMP inhibitors, cyclooxygenase (COX) inhibitors, or sympathetic blockers alleviates IL-17-induced nociception. IL-17 injection also upregulated prostaglandin E2 (PGE-2) creation, MMP-9 activity, and mRNA expression levels of COX-2, MMP-9, and precursor preproendothelin-1 in the synovial membrane. These inflammatory mediators and chemokines play critical roles in the pathogenesis and progression of hyperalgesia response caused by innate and adaptive immunity (56). Taken together, IL-17 is a critical pro-nociceptive cytokine in mBSA-induced arthritis through the promotion of neutrophil migration and production of various pro-inflammatory mediators, such as TNF-α, IL-1β, CXCR1/2 chemokine ligands, MMPs, endothelins, prostaglandins, and sympathetic amines. Nevertheless, the detailed mechanisms remain unclear in these studies. IL-17 may bind to IL-17Rs in nucleus pulposus cells to enhance COX2 expression and PGE2 production *via* the p38/c-Fos and c-Jun N-terminal kinase (JNK/c-Jun) signaling pathways in nucleus pulposus cells to mediate intervertebral disc inflammation and low back pain (57). Based on these data, we can present a reasonable hypothesis: IL-17 activates key signaling pathways to promote the release of pro-nociceptive mediators, cytokines, and inflammatory factors, thereby activating the inflammatory cascade to change neuron sensitivity.

Among these inflammatory factors, TNF has been strongly associated with chronic pain (26) and a close relationship with IL-17 (58). IL-17 may induce hyperalgesia and the effect caused by neutrophil migration, which depend on TNF binding to TNF receptor 1 (TNFR1) (27). TNF has previously been reported to bind to TNFR 1 to mediate hyperalgesia in a CCI model of a mouse sciatic nerve (59). Because TNF-induced hyperalgesia has also been associated with neutrophil migration, IL-17 may cause acute hyperalgesia by promoting TNF release from fibroblasts that stimulates further keratinocyte chemoattractant production, which migrates neutrophil chemotaxis to the plantar tissue and produces analgesic mediators leading to nerve sensitivity. Furthermore, TNFR1 KO and TNF blocking reportedly reduced hyperalgesia caused by IL-17 in an mBAS mice model (39), validating the role of IL-17 and TNF in chronic pain from another perspective. From these results, we can present this hypothesis: IL-17 mediates inflammatory pain by promoting the production of cytokine and inflammatory factors and accumulation of neutrophils and lymphocytes in inflammatory sites.

In addition to promoting inflammatory factor production to mediate chronic pain, studies have also found that IL-17 has a direct effect by binding to IL-17Rs located in neurons. IL-17Rs have been widely expressed in DRG neurons of rats (17, 54). Moreover, IL-17 isoforms can upregulate tetrodotoxin-(TTX-) resistant sodium currents in isolated DRG neurons, implying that IL-17 may play a role in inflammation-evoked sensitization of sensory nociceptive neurons (17). However, the mechanism of the increased TTX- current is still unclear in this study. Another study provides new insight. IL-17 released by astrocytes and IL-17Rs is localized in NR1-immunoreactive neurons and upregulated in chronic pain models. This study also found that IL-17 antibody significantly subsided hyperalgesia and downregulated phosphorylated (p)-NR1 and IL-17RA compared with the control group (29). A body of evidence shows that N-methyl-D-aspartate (NMDA) receptor phosphorylation of neurons is strongly associated with hyperalgesia (60). These studies showed that IL-17 directly acted on nociceptive neurons with NMDA receptors to enhance NMDA receptor phosphorylation to change neuron sensitivity.

## 4 IL-17 and cancer pain

Among cancer patients with bone metastases, 75% experience chronic pain, which can seriously affect quality of life (61, 62). Thus, elucidating the mechanisms of cancer pain and early interventions are urgently needed. IL-17 has been proven important in the initiation and development of immune disorders and cancer (63, 64). Furthermore, a study found that IL-17A levels were negatively associated with clinical pain scores in cancer patients (65). Some studies have also begun to explore the role of IL-17 in the pain of cancer patients with bone metastases. Herein, we will review the potential mechanism of IL-17 in cancer pain.

T cells infiltrated the spinal cord in a rat model of bone cancer pain (BCP) and was accompanied by increased pain and a brief transient rise in regulatory T (Treg) cells, followed by an imbalance of Th17 cells (66). Researchers observed that IL-17 was elevated in both the blood and spinal cord, and IL-17R was upregulated in microglia. Increased production of IL-17/IL-17Rs may further promote microglia activation during BCP development. Furthermore, IL-17-neutralizing antibodies can relieve BCP, as well as suppress Th17/Treg infiltration and inhibit microglial activation. These results suggest that T cells infiltrate the spinal cord accompanied by an imbalance of Th17 cells during BCP progression, thereby promoting IL-17 production which can bind to IL-17R to activate microglial and further increase BCP (66). Thus, targeting infiltrated T cells and increased Th17/Treg ratios in the spinal cord may be a novel therapy for BCP. Further studies of are needed to investigate the role of IL-17 in therapeutic avenues for cancer neuroscience from translational frontiers and clinical opportunities aspects.

## 5 Direct and indirect mechanisms of IL-17 in chronic pain

**Figure 1:**
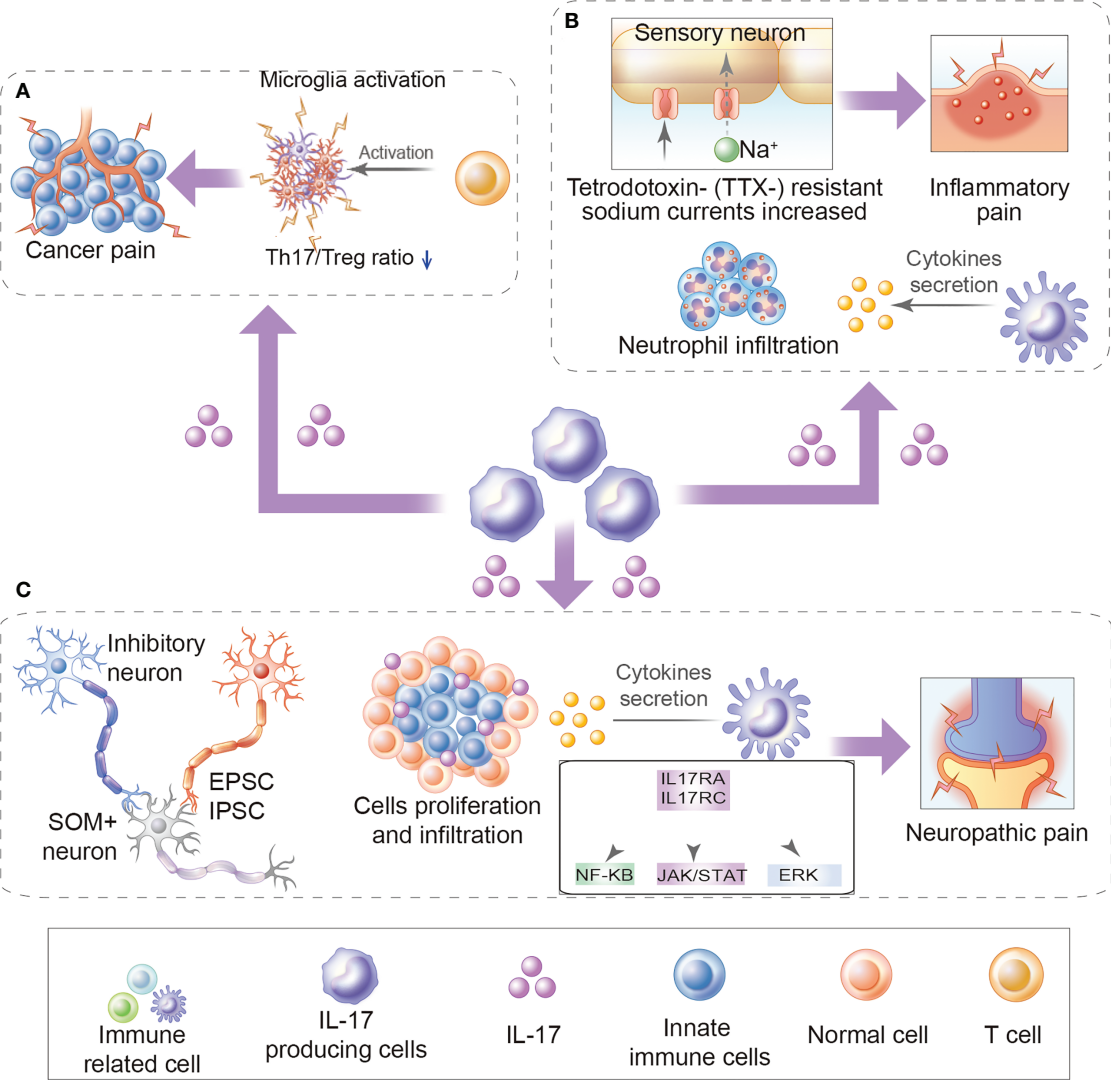
Role of IL-17 in chronic pain. **(A)** The role of IL-17 in cancer pain: For example, increased production of IL-17/IL-17Rs may further promote Th17/Treg infiltration and microglia activation during bone cancer pain development. **(B)** Inflammatory pain: For example, IL-17 mediates inflammatory pain by promoting the production of cytokine and inflammatory factors and accumulation of neutrophils and lymphocytes in inflammatory sites. Besides, IL-17 upregulates TTX- resistant sodium currents in isolated DRG neurons. **(C)** Neuropathic pain: For example, IL-17/IL17Rs regulate EPSCs and IPSCs, promote cytokine production, immune cell activation, and immune cell proliferation and infiltration, activates ERK and NF-κB, and JAK/STAT signal pathways.

The above review respectively elaborates on the potential role of IL-17 in chronic pain from different diseases (**Figure 1** and **Table 1**). However, reviews that comprehensively summarize the mechanistic role of IL-17 in chronic pain are lacking. Herein, we separate the mechanistic roles of IL-17 in chronic pain into direct (IL-17 directly binds to neuronal IL-17R to induce chronic pain) and indirect roles (IL-17 induces chronic pain by regulating infiltrating immune cells and pain mediator production).

**Table 1:** Summary of characteristics of studies on the role of IL-17 in chronic pain.

Pain type	Animal model	Animal sex	Expression level	Mechanism	PMID
Neuropathic Pain	Sciatic nerve ligation	Male	Upregulation	IL-17 promotes infiltration of T cells and macrophages and activation of microglia and astrocytes in injured nerve	20889388
	Sciatic nerve ligation	Male	Upregulation	IL-17 promotes inflammatory cell infiltration and pro-inflammatory cytokine (TNF-α, IL-6, and IFN-c) in damaged nerves	24721689
	Sciatic nerve ligation	Male	Upregulation	IL-17 activated CaMKII/CREB Signaling in Spinal Neurons	26166359
	Spinal nerve ligation	Male	Upregulation	IL-17 promoted proliferation of astrocytes and secretion of proinflammatory cytokines	27959414
	Chronic constriction injury	Female	Upregulation	MMP-2/9 inhibitors decreased the levels of IL-17 and TNF-α	22676642
	Chronic constriction injury	Female	Upregulation	FN-CS1 contributes to mechanical pain hypersensitivity by increasing IL-17-expressing (presumably, Th17) cells.	26337825
	Spinal cord injury	Male	Upregulation	IL-17 promotes spinal cord neuroinflammation *via* activation of the transcription factor STAT3	24914249
	Spinal cord injury	Male	Upregulation	CCL20 aggravates neuroinflammation *via* regulation of Th17 cell recruitment and IL-17 level	27334337
	Spinal cord injury	Male	Upregulation	IL-17 induces reactive astrocytes and activates IL-17-JAK/STAT-VEGF axis	28281545
	Chemotherapy-induced neuropathic pain	Male	Upregulation	IL-17 enhances excitatory postsynaptic currents and reduces inhibitory postsynaptic currents	31747607
Inflammatory pain	Incubation of cultured DRG neurons with either TNF-α or IL-1ß	Male	Upregulation	IL-17 increases ERK and NF-κB, and upregulate TRPV4	23147107
	Antigen (mBSA)-induced arthritis	Male	Upregulation	IL-17 upregulates TNF-α, IL-1b, CXCL1, MMPs, endothelins, prostaglandins, sympathetic amines	19969421
	Low back pain	/	Upregulation	IL-17 activates p38/c-Fos and JNK/c-Jun signal pathways	26988982
	Hyperalgesia in mice model induced by injection of recombinant IL-17 or TNF	Male	–	IL-17 promotes TNF/TNFR1 and neutrophil infiltration	21507574
	Antigen (mBSA)-induced arthritis	Male/Female	Upregulation	IL-17 upregulates TTX- resistant sodium currents	28871176
	Complete Freund adjuvant injection	Male	Upregulation	IL-17 promotes NMDA NR1 phosphorylation	23246025
	Antigen-induced arthritis	Male	Upregulation	IL-17 promote phosphorylation of protein kinase B and ERK	23192794
Cancer pain	Bone cancer pain	Male	Upregulation	IL-17 activates microglial	30685532

IL-17, interleukin-17; TNF-α, Tumor necrosis factor-α; IFN-c, interferon-c; IL-6, interleukin-6; CaMKII, Calcium/calmodulin-dependent protein kinase II; CREB, cAMP-response element binding protein; MMP2/9, Matrix metalloproteinase2/9; STAT, signal transducer and activator of transcription; CCL20, Chemokine (C-C motif) ligand 20; JAK, Janus kinase; VEGF, vascular endothelial growth factor; FN-CS1, Fibronectin- connecting segment 1; ERK, Extracellular signal-regulated kinase; NF-κB, nuclear factor-kappaB; TRPV4, transient receptor potential vanilloid 4; IL-1beta: interleukin-1beta; JNK, Jun amino terminal kinase; TTX-, tetrodotoxin-; NMDA, N-methyl-D-aspartate.

### 5.1 Direct role of IL-17 in chronic pain

**Figure 2:**
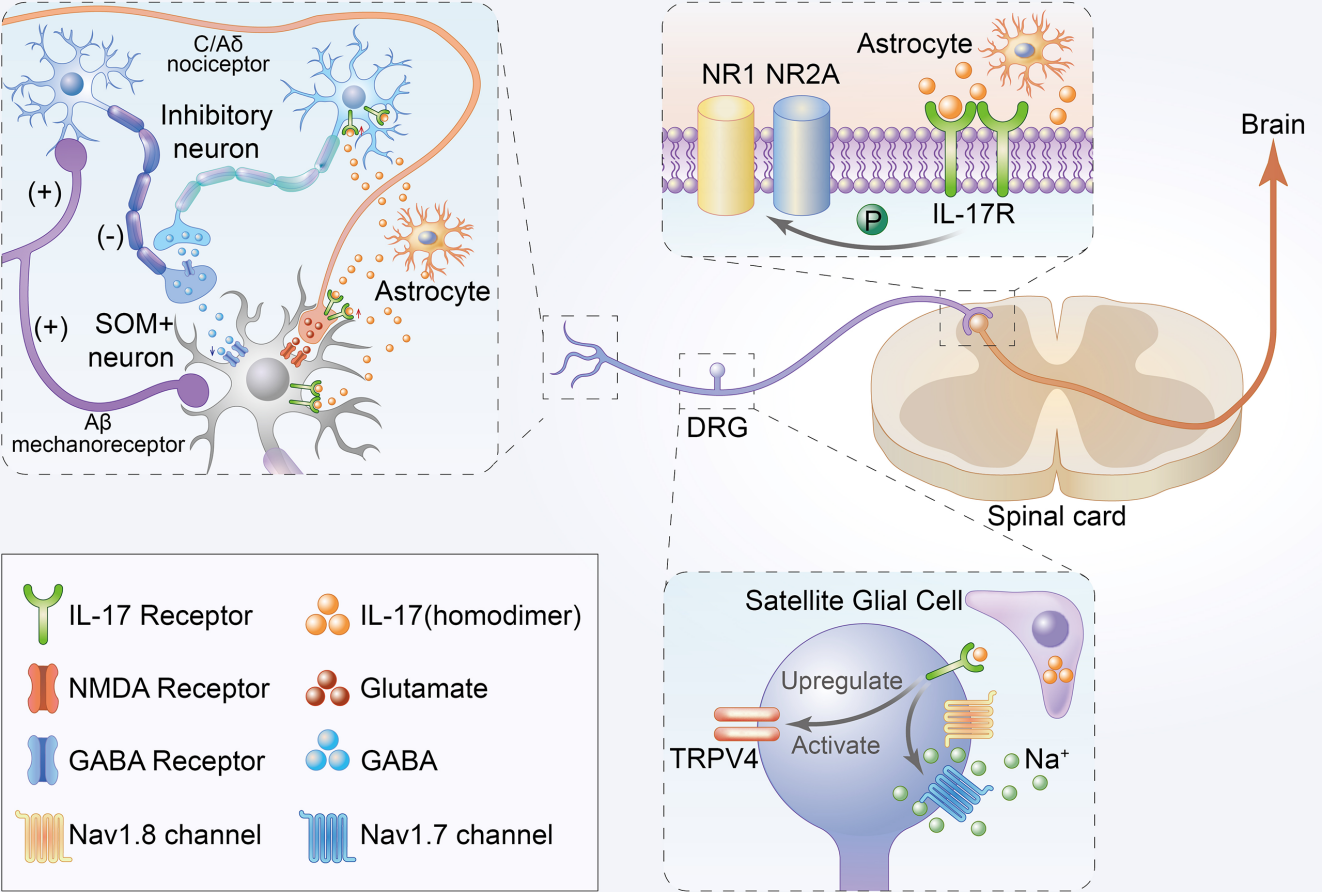
Schematic illustration of direct mechanisms of IL-17 in the development of chronic pain. Firstly, IL-17 produced by astrocytes not only enhances EPSCs but also suppresses inhibitory IPSCs and GABA-induced currents in the lamina II by interacting with neurons. Moreover, IL-17 from astrocytes can directly bind to IL-17Rs to mediate NAMD NR1 phosphorylation in the rat spinal cord. Thirdly, IL-17 from satellite glial cell can directly bind to IL-17R to upregulate and activate TRPV4 and increase tetrodotoxin-sensitive sodium currents in DRG neurons.

IL-17 was mainly expressed in DRG satellite glial cells. In contrast, IL-17Rs were found to be widely expressed in small-sized mouse DRG IB4+ and CGRP+ neurons with a predominance in IB4+ neurons, although not in large-sized NF-200+ DRG neurons (17, 18). The respective localization of IL-17/IL-17R signaling offers a cellular basis for neuron-glial interaction and promotes both central and peripheral sensitization *via* the central and peripheral nervous systems. Here, we summarize the direct role of IL-17 in chronic pain (**Figure 2**).

IL-17/IL-17Rs mediate neuron-glial interactions and neuronal hyperexcitability in chemotherapy-induced chronic pain (18). IL-17 produced by astrocytes not only enhances EPSCs but also suppresses inhibitory IPSCs and GABA-induced currents in the lamina II by interacting with neurons. Increased EPSCs and decreased IPSCs are reportedly associated with central sensitization and play key roles in the mechanisms of chronic pain (67, 68). Moreover, IL-17 is released by astrocytes, and IL-17R is localized in NR1-immunoreactive neurons with decreased p-NR1 and upregulated in chronic pain models (29). Some studies have established that hyperalgesia correlates with enhanced NR1 phosphorylation in the rat spinal cord (69).

IL-17 isoforms increased TTX- resistant sodium currents in isolated DRG neurons (17). TTX- resistant sodium currents were confirmed to be associated with chronic pain, and TTX- was also considered a potential therapeutic agent for pain (70). Remarkably, isolated and cultured rat DRG neurons showed significant TRPV4 upregulation upon long-term exposure to IL-17A (38), which is considered a candidate transduction molecule for mechanical hyperalgesia (71). TRPV4 activation was also associated with tetrodotoxin-sensitive sodium currents (72). In summary, the direct role of IL-17 in chronic pain may occur through binding with IL-17Rs located in neurons, thereby regulating the corresponding sodium currents in neurons.

### 5.2 Indirect role of IL-17 in chronic pain

**Figure 3:**
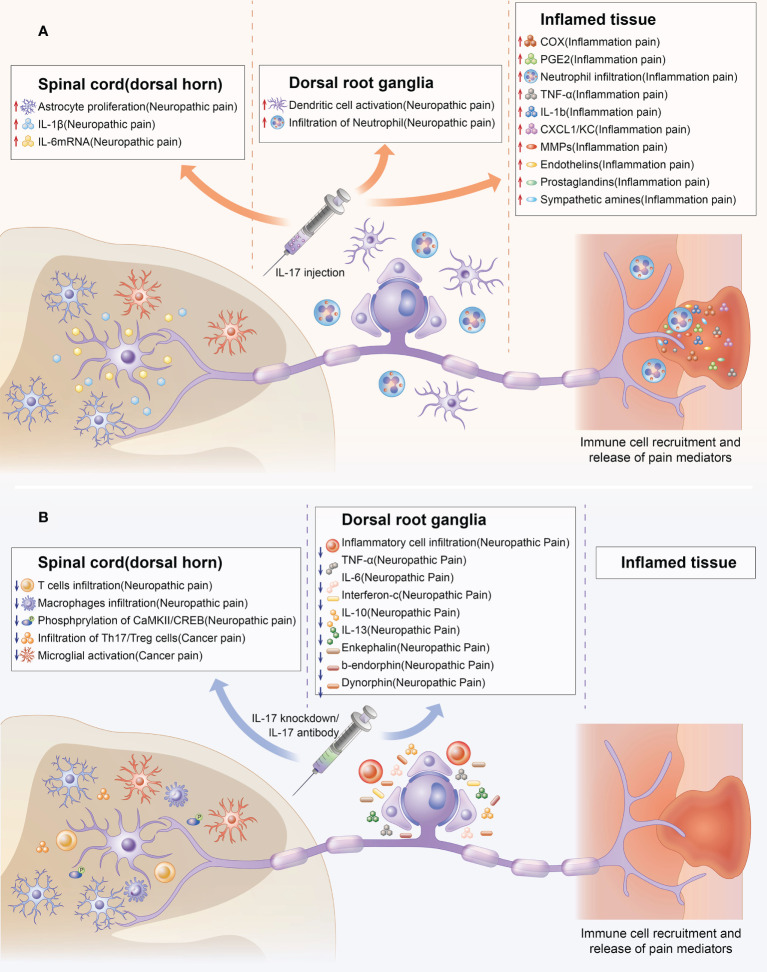
Indirect effect of IL-17 in chronic pain. Upregulation and downregulation of IL-17 can mediate chronic pain through chemical signals at several steps of nociceptive transmission. The colored boxes summarize the effects of IL-17 injection and IL-17 knockdown in the preclinical pain models in which they were described. IL-17 can mediate chronic pain by promoting the production of pain factors (such as IL-6,TNF-α, PGE2) and activation of cells (such as astrocyte, microglial, T cells, macrophage infiltration) related to chronic pain from spinal cord, DRG, and inflamed tissue levels. **(A)** Indirect effect of IL-17 upregulation in chronic pain. **(B)** Indirect effects of IL-17 knockdown in chronic pain.

Aside from the direct mechanisms, there are also important indirect mechanisms. IL-17 can mediate chronic pain by promoting the production of pain factors and activation of cells related to chronic pain (**Figure 3**). Microglia reportedly play an important role in chronic pain (73). IL-17 and IL-17R levels increased with microglia activation during BCP development (66). Furthermore, IL-17 KO decreased T cell and macrophage infiltration to injured sciatic nerves and microglia and astrocyte activation in the spinal cord (36). These cells were also strongly associated with chronic pain (74, 75).

Aside from activating immune cell-related chronic pain, IL-17 can also mediate chronic pain by inducing the production of other pain-related factors. IL-17 production was enhanced in a pSNL model and accompanied by significant upregulation of mRNA levels of IL−1β and IL−6. IL-1β and IL-6 are important in chronic pain (53, 76). Furthermore, IL-17 knockdown may decrease pro-inflammatory cytokines (TNF-α, IL-6, and interferon-γ) levels in damaged nerves (37). TNF-α and interferon-γ have also been confirmed to be closely related to chronic pain (77, 78). IL-17 injection into joints increased TNF-α, IL-1β, CXCL1, and PGE-2 production; MMP-9 and COX-2 activity; and MMP-9 mRNA expression in the synovial membrane (39). The inflammatory process has also been associated with chronic pain development (79–81). Furthermore, IL-17A enhanced COX2 expression and PGE-2 production *via* the p38/c-Fos and JNK/c-Jun signaling pathways. Moreover, IL-17A KO significantly reduced p-CaMKII and p-CREB levels, which have been associated with hyperalgesia and central sensitization in the spinal cord (42, 82, 83).

## 6 Promising target for chronic pain: IL-17

IL-17 has been associated with chronic pain in not only chronic pain animal models, but also in clinical patients. Th17 lymphocyte and IL-17 levels in lumbar disc herniation were positively correlated with visual analog scale scores recorded for preoperative pain intensity (84). The levels of IL-17A mRNA and protein expression in patients with bladder pain syndrome type 3C were higher than those in healthy volunteers (85). Furthermore, IL-17 levels had a positive correlation with pain severity in patients with osteonecrosis of the femoral head (86). In addition, IL-17 levels were negatively associated with clinical pain scores in patients with cancer (65). Interestingly, opioid requirements were increased in patients with the AA genotype of rs2275913 SNP of IL-17A during and after surgery (87).

IL-17/IL-17R upregulation was observed in rodent models of NP and patients with pain in many studies, and these data demonstrate that IL-17/IL-17R antibodies or IL-17-knockdown can effectively attenuate chronic pain (18, 42, 60). Hence, there are certainly reasons to believe that targeting IL-17/IL-17R signaling is a promising treatment for chronic pain. In addition, IL-17/IL-17R antibodies can relieve and cure autoimmune diseases (88) such as rheumatoid arthritis (89) and psoriatic disease (90).

Currently, IL-17/IL-17R blockers have been developed to treat inflammatory disease. Brodalumab (Kyntheum^®^) is a human anti-IL-17RA monoclonal antibody available for use in patients with moderate to severe psoriasis (91). Furthermore, brodalumab was associated with significant improvement in signs and symptoms of psoriatic arthritis versus placebo (92). Researchers found that mouse IL-17RA antibody was effective in suppressing neuronal hyperexcitability after paclitaxel, which might indicate brodalumab could be used to treat CIPN and neuropathic pain (18). While, Secukinumab (Cosentyx^™^) is the first fully human monoclonal immunoglobulin G1κ antibody targeting human IL-17A, with indications for psoriasis, psoriatic arthritis, and ankylosing spondylitis (93–95). Ixekizumab, a humanized immunoglobulin G4 variant IL-17A-neutralizing antibody can bind human and cynomolgus monkey IL-17A with high affinity to inhibit interactions between IL-17A and its receptors in binding assays and potently blocks IL-17A-induced GRO or KC secretion in cell-based assays (96). Ixekizumab has been widely applied to patients with psoriasis and psoriatic arthritis (97, 98). We summarized the stages and clinical trial results for drugs targeting IL-17/IL-17Rs in various diseases in **Table 2**. Thus, the proposal of drugs targeting IL-17/IL-17Rs is plausible for patients with pain. Although direct clinical trial evidence for these drugs in the treatment of chronic pain is lacking, some studies have demonstrated that IL-17/IL-17R blockers can relieve painful symptoms in patients (99). Secukinumab-treated patients had rapid improvement in pain and fatigue scores in an overall population after 1 and 4 weeks, respectively, indicating the potential of IL-17/IL-17R blockers for pain management.

**Table 2:** Summary of IL-17/IL-17R drug targeting in various diseases.

Target	Drug name	Indication	Therapeutic efficacy	Clinical Experimental stage	Primary endpoint	Interventions	Number of patients	PMID	Identifier
IL-17R	Brodalumab	Psoriasis	Yes	III	Skin clearance efficacy at week 120	Brodalumab 210 mg Q2W after Ustekinumab n=274; Continuous Brodalumab 210 mg Q2W n=168; All patients who received any dose of Brodalumab n=1790	2232	31175909	NCT01708603
IL-17R	Brodalumab	Psoriatic arthritis	Yes	III	ACR20 response at week 16.	Brodalumab 140mg(N=318); Brodalumab 210mg (N=322); Placebo (N=322)	962	33106286	NCT02029495 NCT02024646
IL-17R	Brodalumab	Methotrexate-resistant rheumatoid arthritis	No	Ib	ACR20 at week 13	SC:50mg,140mg,210mg(n=18); IV:420mg,700mg(n=12) Placebo(n=10)	40	24286136	NCT00771030
IL-17R	Brodalumab	Moderate-to-Severe Crohn's Disease	No	II	CDAI remission (≤150) at week 6	Brodalumab Q4W 210mg (N=32); 350mg (N=33); 700 mg (N=33); Placebo (N=32)	130	27481309	No.
IL-17R	Brodalumab	Moderate to severe asthm	No	No	ACQ score to week 12	Brodalumab Q2W 140mg (N=74); 210mg (N=76); 280 mg (N=76) Placebo (N=76)	302	24200404	NCT01199289
IL-17	Secukinumab	Plaque psoriasis	Yes	III(ERASURE)	PASI 75 at week 12	Secukinumab, 300mg (n=245); 150mg(n=245); Placebo(n=248)	738	25007392	No.
IL-17	Secukinumab	Psoriatic arthritis	Yes	III	ACR20 at week 24	Subcutaneous Secukinumab; 300 mg (n=100); 150 mg (n=100); 75 mg (n=99); Placebo (n=98)	397	26135703	NCT01752634
IL-17	Secukinumab	Ankylosing Spondylitis	Yes	III (MEASURE 1)	ASAS 20 response criteria at week 16.	Secukinumab 150 mg SC (n=125); Secukinumab 75 mg SC (n=124); Placebo (n=122)	371	26699169	NCT01358175
				III (MEASURE 2)		secukinumab 150 mg SC (n=72) secukinumab 75 mg SC (n=73) placebo (n=74)	219		NCT01649375
IL-17	Secukinumab	Rheumatoid Arthritis	Yes	III	ACR20 response	Secukinumab 150 mg (n=137); Secukinumab 75 mg (n=138); Abatacept (n=138); Placebo (n=138);	551	28217871	No
IL-17	Secukinumab	Noninfectious uveitis	No	III SHIELD	Recurrence rate at 24 weeks	Secukinumab 300 mg (loading, then q2w), n = 32 Secukinumab 300 mg (loading, then q4w), n = 31 Placebo ((loading, then q2w), n = 34	97	23290985	NCT00995709
				INSURE	Vitreous haze score at week 28 or at the time of rescue	Secukinumab 300 mg (loading, then q2w), n = 8 Secukinumab 300 mg (loading, then q4w), n = 10 Secukinumab 150 mg (loading, then q4w), n = 8 Placebo ((loading, then q2w), n = 5	31		NCT01095250
				ENDURE	Time to first recurrence of active intermediate uveitis, posterior uveitis, or panuveitis in either eye from baseline to week 24.	Secukinumab 300 mg (loading, then q2w), n = 21 Secukinumab 300 mg (loading, then q4w), n = 20 Secukinumab 150 mg (loading, then q4w), n = 24 Placebo ((loading, then q2w), n = 27	92		NCT01032915
IL-17	Secukinumab	Moderate to severe Crohn's disease	No	III	CDAI scores at week 6.	Secukinumab 2*10 mg/kg, n=39 Placebo,n=20	59	22595313	NCT01009281
IL-17	Ixekiumab	Moderate-to-Severe Plaque Psoriasis	Yes	III UNCOVER-1	PASI 75 at week 12.	Ixekizumab Every 4 wk (N=432) Ixekizumab Every 2 wk (N=433) Placebo (N=431)	1296	27299809	NCT01474512
				UNCOVER-2		Ixekizumab Every 2 wk (N=351) Ixekizumab Every 4 wk (N=347) Etanercept (N=358) Placebo (N=168)	1224		NCT01597245
				UNCOVER-3		Ixekizumab Every 4 wk (N=386) Ixekizumab Every 2 wk (N=385) Etanercept (N=382) Placebo (N=193)	1346		NCT01646177
IL-17	Ixekiumab	Psoriatic arthritis	Yes	III	ACR 20 response at week 24	IXEQ2W N=103 IXEQ4W N=107 Adalimumab 40 mg Q2W N=101 Placebo N=106	417	27553214	NCT01695239 EudraCT2011-002326-49
IL-17	Ixekiumab	Non-radiographic axial spondyloarthritis	Yes	III	ASAS 40 response at weeks 16 and 52	Ixekizumab Q2W group (n=102) Ixekizumab Q4W group (n=96) Placebo group (n=105)	303	31813637	NCT02757352

SC, subcutaneous; IV, intravenous; ACR20, American College of Rheumatology 20; CDAI, Crohn’s disease activity index; ACQ, Asthma Control Questionnaire; ASAS 20, Assessment of Spondyloarthritis International Society; PASI, Psoriasis Area and Severity Index.

## 7 Concluding remarks and future perspective

Although great progress has been made in pain research over the past decade, there has been little translation of preclinical results into clinical practice. Currently, patients are frequently undertreated, and more effective analgesic drugs are urgently needed. Previous studies have shown that IL17 plays a critical role in the pathogenesis of neuropathic pain, inflammatory pain and cancer pain (**Figure 1**). Increased expression IL17 levels have been observed in various chronic pain models (**Table 1**). Notably, treatment with IL-17/IL-17R antibodies or IL-17-knockdown considerably attenuated chronic pain and relieve autoimmune diseases (**Table 2**). While reviewing the current evidence, we have discussed the direct and indirect relationship between IL17 and chronic pain (**Figures 2**, **3**). IL-17/IL-17R blockers may be a novel and beneficial therapeutic tool for chronic pain management. However, these findings raise additional questions.

First, whether IL-17 blockers exert similar analgesic effects on other types of chronic pain (such as herpetic neuralgia and diabetes peripheral neuropathy) requires further investigation. Each type of chronic pain has unique characteristics. However, they may have the same pathogenesis. Therefore, it is important to reveal the beneficial effects of IL-17 inducers in other types of chronic pain.

Second, current studies have mostly focused on the analgesic effects of IL-17 blockers rather than the underlying mechanisms. For example, IL-17 exerted a limited role in the acute nerve injury phase and associated acute pain, while mainly playing a vital role in the later stages of nerve injury in the development of NP. Therefore, the detailed mechanisms underlying the analgesic effects of IL-17 blockers and the mechanisms by which so‐called IL-17 blockers repress IL-17 need to be elucidated. Recently, Secukinumab (IL-17 antibody)-treated patients showed rapid improvement in pain and fatigue scores in an overall population. The specific IL-17/IL-17R blockers in preclinical studies and clinical trials for autoimmune diseases and chronic pain area are further studies warranted.

Finally, current studies have primarily focused on the relationship between IL-17 (IL-17A) and chronic pain. However, other IL-17 family members (e.g., IL-17B, and IL17-F) are also involved in the mechanisms of chronic pain. Therefore, the involvement of additional members of the IL-17 family in chronic pain could be reported in the future. Large‐scale, multicenter, prospective clinical trials are needed. Given the analgesic effects of IL-17 blockers, efforts to discover more IL-17 blockers would have a large impact on clinical and public health.

## Author contributions

XJJ, RHZ, and YJZ were the major contributors in writing and revising the manuscript. YJZ, QL and WYZ performed the literature search. XJJ, RHZ, and TZ discussed and revised the manuscript. QL and WYZ participated in the design of the review and helped to finalize the manuscript. All authors read and approved the final manuscript

## Acknowledgments

We would like to thank Editage (www.editage.cn) for English language editing.

## Conflict of interest

The authors declare that the research was conducted in the absence of any commercial or financial relationships that could be construed as a potential conflict of interest.

## Publisher’s note

All claims expressed in this article are solely those of the authors and do not necessarily represent those of their affiliated organizations, or those of the publisher, the editors and the reviewers. Any product that may be evaluated in this article, or claim that may be made by its manufacturer, is not guaranteed or endorsed by the publisher.
